# Exploring mechanisms governing cartilage interstitial fluid load support in lubrication through experimental and computational analysis

**DOI:** 10.1038/s41598-026-41939-9

**Published:** 2026-03-10

**Authors:** Janne T. A. Mäkelä, Taylor B. Lawson, Rami K. Korhonen, Mark W. Grinstaff, Brian D. Snyder

**Affiliations:** 1 Center for Advanced Orthopaedic Studies, Beth Israel Deaconess Medical Center, Harvard Medical School, Boston, MA USA; 2 Department of Chemistry, Boston University, Boston, MA USA; 3 Department of Technical Physics, University of Eastern Finland, Kuopio, Finland; 4 Department of Biomedical Engineering, Boston University, Boston, MA USA; 5 Department of Mechanical Engineering, Boston University, Boston, MA USA; 6 Department of Medicine, Boston University, Boston, MA USA; 7 Department of Orthopaedic Surgery, Boston Children’s Hospital, Harvard Medical School, Boston, MA USA

**Keywords:** Engineering, Materials science

## Abstract

Articular cartilage enables nearly frictionless joint motion by regulating how interstitial fluid pressurizes and supports mechanical loads. Loss of this function is a key contributor to osteoarthritis, yet the mechanisms linking tissue composition, fluid pressurization, and lubrication remain poorly understood. Here we combine tribological testing of healthy and enzymatically degraded bovine cartilage with sample-specific finite element (FE) modeling to investigate how interstitial fluid load support (IFLS) governs the coefficient of friction (COF). While direct measurement of IFLS is technically demanding, our FE-based approach enables empirical estimation of IFLS. Cartilage plugs were articulated against each other under creep loading and lubricated with either synovial fluid or saline. The finite element model enabled empirical quantification of IFLS during testing, which we validated against previously reported experimental data. We observed that once interstitial fluid load support declined to low levels, lubrication became increasingly dependent on the external medium. While the strain–IFLS relationship remained linear across all conditions, degeneration disrupted the otherwise consistent IFLS–COF relationship. These findings help clarify how fluid pressurization and tissue integrity jointly regulate cartilage lubrication, providing a functional framework for evaluating cartilage repair strategies and extending to other biphasic materials such as hydrogels and engineered tissues.

**Supplementary Information:**

The online version contains supplementary material available at 10.1038/s41598-026-41939-9.

## Introduction

Hyaline cartilage dissipates loads and provides a smooth, nearly frictionless gliding articular surface in diarthrodial joints. It is a biphasic porous tissue comprised of a collagen fibril network that provides structure and tensile strength, complemented by a matrix of negatively charged polysaccharides, glycosaminoglycans (GAGs), together regulating interstitial fluid transport^1^. The diffusing drag of interstitial fluid flowing through the porous network accounts for the tissue’s non-linear viscoelastic behavior, subject to the strain-dependent permeability of the tissue ^2^, a classical concept established decades ago and still foundational today, although later complemented by evidence of intrinsic fibrillar viscoelasticity^3^.

Hyaline cartilage progressively breaks down in osteoarthritis (OA), which manifests itself clinically by pain and decreased joint function, disabling hundreds of millions of individuals worldwide. The etiology of OA is multi-factorial, often induced by mechanical injury as a consequence of trauma, joint instability, ligamentous deficiency, skeletal malalignment, obesity or anatomic deformity. Mechanical overloading induces surface wear and superficial zone delamination^4^ that propagates into structural failure: fissuring/fibrillation^5^, tissue swelling^6^, compositional loss of GAG^7,8^, and derangement of the collagen network^9^. The depletion of cartilage GAG and subsequent collagen network degradation increases hydraulic permeability and decreases cartilage stiffness^8^. Concomitant with the pathophysiological processes affecting cartilage composition, structure, and function, is a decline in synovial fluid lubricity, owing to a decrease in the concentration and molecular weight of hyaluronic acid, which diminish its rheological and tribological properties^10^.

Regeneration of functional hyaline cartilage remains a challenge. To address the biomechanical consequences of cartilage injury, strategies to employ biomaterials and chemical crosslinking methods, e.g., hyaluronic acid or poly(ethylene glycol) to fortify or protect the tissue have shown improvements in biphasic mechanical properties^11–13^. Recent efforts have focused on reinforcing or resurfacing the hydrophilic extracellular matrix (ECM) to restore essential cartilage material properties^11,13–15^. Strengthening the solid phase of cartilage has been shown to influence the fluid phase, potentially reestablishing the low permeability characteristic of healthy cartilage. These structural modifications may contribute to addressing key biomechanical challenges associated with OA, a condition that significantly impacts joint function and quality of life.

At the heart of cartilage functionality, i.e. interplay between the solid and fluid phases, lies the concept of Interstitial Fluid Load Support (IFLS). This fraction, *W*_p_/*W*, reflects the degree to which the applied load (*W*) is carried by the interstitial fluid (*W*_p_). Compression deforms the porous network, reduces pore volume, and increases interstitial fluid pressure. This orchestrated interplay allows the entrapped interstitial fluid to support over 90% of the applied joint load^13,16,17^.

Despite the pivotal role IFLS plays, a gap exists in our understanding of how loading, tissue properties, and the friction–IFLS relationship evolve during tissue degradation. We hypothesize that IFLS is the principal mechanistic link between tissue composition, creep deformation, and friction, and that enzymatically induced matrix degeneration would disrupt this otherwise robust relationship. To investigate these mechanisms, we employ a tribo-rheometer configuration^18^ to quantify cartilage-on-cartilage friction across varying IFLS levels using healthy and enzymatically degraded bovine osteochondral plugs (mimicking Outerbridge grade 0–1) lubricated with either bovine synovial fluid or saline (a surrogate for OA synovial fluid). Complementing the experimental measurements, we use a static creep axisymmetric fibril-reinforced poroviscoelastic finite element (FE) model^3,19,20^, which captures the experimental axial deformation and enables empirical derivation of IFLS during loading. By combining creep-based tribological testing with FE-based estimation of tissue pressurization, our approach provides both realistic frictional responses and quantitative insight into internal fluid–solid interactions that cannot be measured directly.

Our primary goal is twofold: first, to examine how articulation velocity influences the friction–IFLS relationship under different lubricant conditions, and second, to determine how matrix degradation alters the fundamental function of cartilage. This multifaceted framework integrates loading characteristics, lubricant environment, and evolving IFLS dynamics within the fibril-reinforced poroviscoelastic material, offering new insight into cartilage structure–function relationships. In contrast to previous work that examined IFLS under simplified geometries or cartilage–counterface configurations, our approach evaluates IFLS dynamically during true cartilage-on-cartilage articulation and compares how articulation velocity and controlled proteoglycan depletion influence the IFLS–friction relationship across distinct lubricant environments.

## Results

**Fig. 1:**
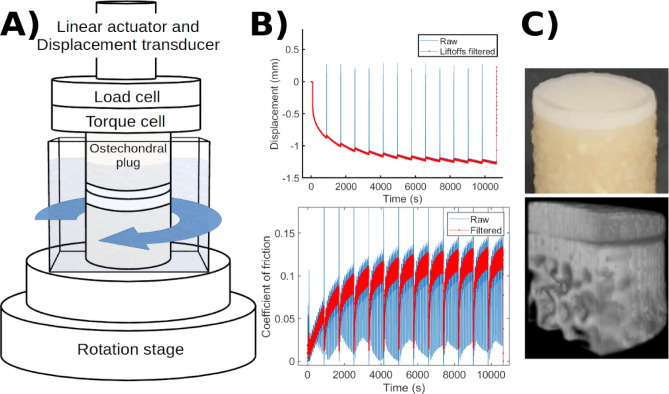
Tribology measuring setup and representative signals. (**A**) Tests were conducted on a BOSE Electroforce 3200 with in-line axial and torsional load cells. (**B**) Apposing cartilage surfaces of paired, coaxially aligned specimens submerged in lubricant were tested at a constant compressive stress of 0.78 MPa and axial displacement was recorded (top). The corresponding coefficient of friction (bottom) was calculated from the measured torque; intermittent lift-offs were filtered prior to analysis as described in the Methods.

The tribological test configuration is shown in Fig. 1, where paired osteochondral plugs were articulated under controlled compression and torsion.

### FE validity, creep deformation and IFLS

**Fig. 2:**
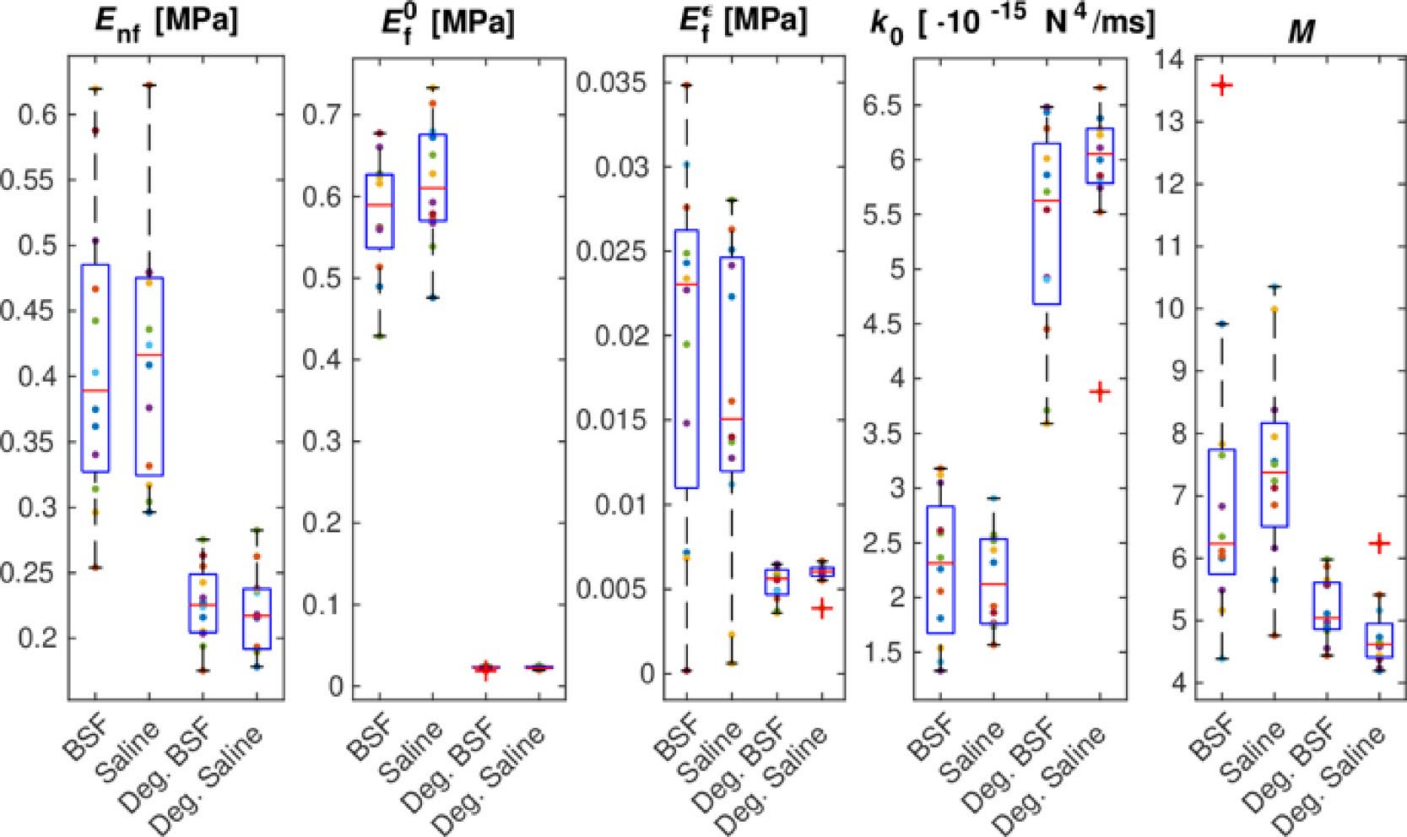
Acquired model parameters from the experiments: nonfibrillar matrix modulus (E_nf_), initial and strain-dependent fibril network modulus (*E*_f_^0^, *E*_f_^*ε*^, respectively), initial permeability (*k*_0_) and strain-dependency of permeability (*M*). BSF = bovine synovial fluid, Deg.=degraded, i.e., Chondroitinase treated group.

Based on µCT images, average cartilage thicknesses were 1.71 ± 0.19 mm (BSF), 1.72 ± 0.33 mm (saline), 2.08 ± 0.69 mm (chondroitinase + saline), and 2.20 ± 0.61 mm (chondroitinase + BSF). Although the degraded groups showed slightly higher mean thickness, the differences were not statistically significant (*p* > 0.05). Creep deformation, derived from the FE model, accurately replicated the experimental data (*R*² = 0.995 ± 0.004, Supplementary Fig. 1). Replication accuracy was comparable between healthy and degraded groups, confirming that omitting sample-specific architecture did not affect the validity of the analysis. The derived model parameters, initial fibril network modulus, *E*_f_^0^, strain-dependent fibril network modulus, *E*_f_^ε^, and nonfibrillar matrix modulus, *E*_nf_, were markedly lower for the degraded chondral specimens compared to the healthy cartilage (Fig. 2). Correspondingly, the initial permeability, *k*_0_, was increased for the degraded specimens.

**Fig. 3:**
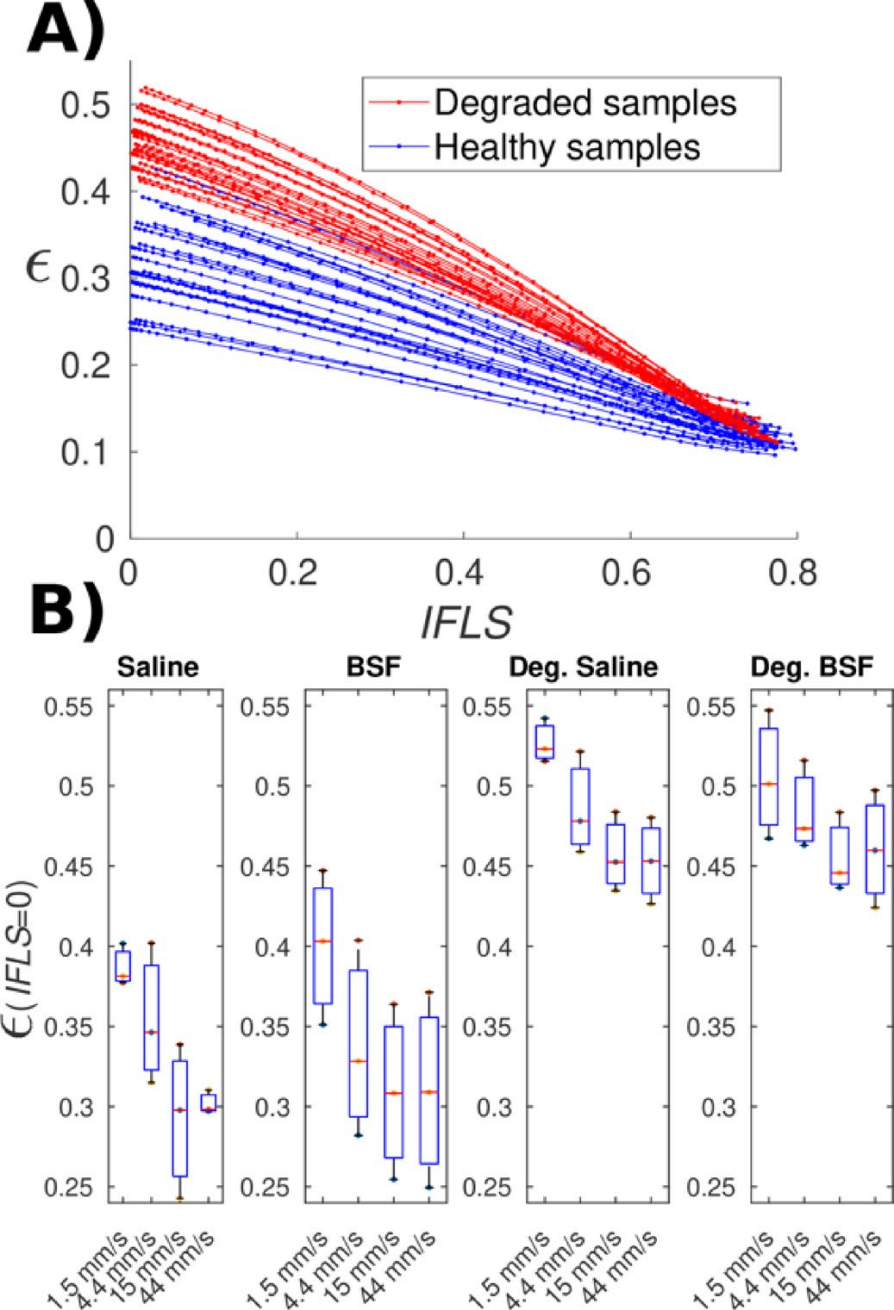
Axial strain (*ε*) as a function of Interstitial fluid load support (IFLS). (**A**) Chondroitinase treated samples marked with red. Healthy samples marked with blue. (**B**) Estimated axial strains when IFLS = 0 for different effective articulation velocities of the baths. Kruskal–Wallis testing showed a significant effect of tissue integrity on *ε*(0) (*p* < 0.0001), whereas lubricant had no effect; articulation velocity showed a modest effect (*p* = 0.03). BSF = bovine synovial fluid, Deg.=degraded, i.e., Chondroitinase treated group.

During the creep phase, the average fluid load support decreased from 80 ± 2% to 1 ± 1% (Fig. 3A). The FE derived IFLS accounted for 99% of the variation in axial strain, *ε*, during creep deformation (*R*^2^ = 0.997 ± 0.002):

*ε* = (-0.29 ± 0.057)·IFLS + (0.33 ± 0.05),

*ε* = (-0.29 ± 0.065)·IFLS + (0.34 ± 0.06),

*ε* = (-0.46 ± 0.050)·IFLS + (0.48 ± 0.038),

*ε* = (-0.46 ± 0.046)·IFLS + (0.48 ± 0.035),

for the healthy saline, healthy bovine synovial fluid (BSF), degraded saline, and degraded BSF samples, respectively.

Tissue integrity (healthy vs. degraded) affected the relationship between axial strain and IFLS: the maximum strain at IFLS = 0, *ε*(0), was 48 ± 4% and 34 ± 5% for the degraded and healthy samples, respectively (K-W *p* < 0.0001). The type of lubricant (saline or BSF) did not affect the experimental axial strain values or the *ε* vs. IFLS relationship, but the articulation velocity did affect the strain values (*p* = 0.03, Fig. 3B).

### COF, IFLS, articulation velocity and lubricant viscosity

**Fig. 4:**
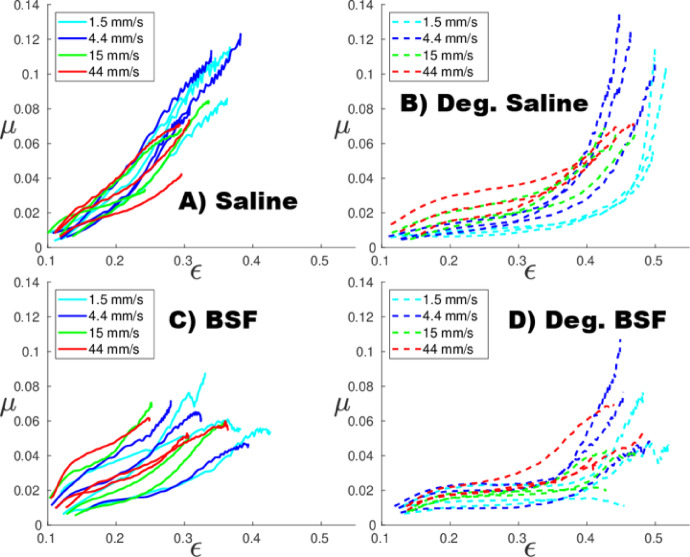
Experimentally measured coefficient of friction (*µ*) as a function of axial strain (*ε*) at different effective velocities (*v*_eff_=1.5, 4.4, 15, 44 m/s). (**A**) Healthy plugs in saline, (**B**) degraded plugs in saline, (**C**) healthy plugs in bovine synovial fluid, and (**D**) degraded plugs in bovine synovial fluid.

**Fig. 5:**
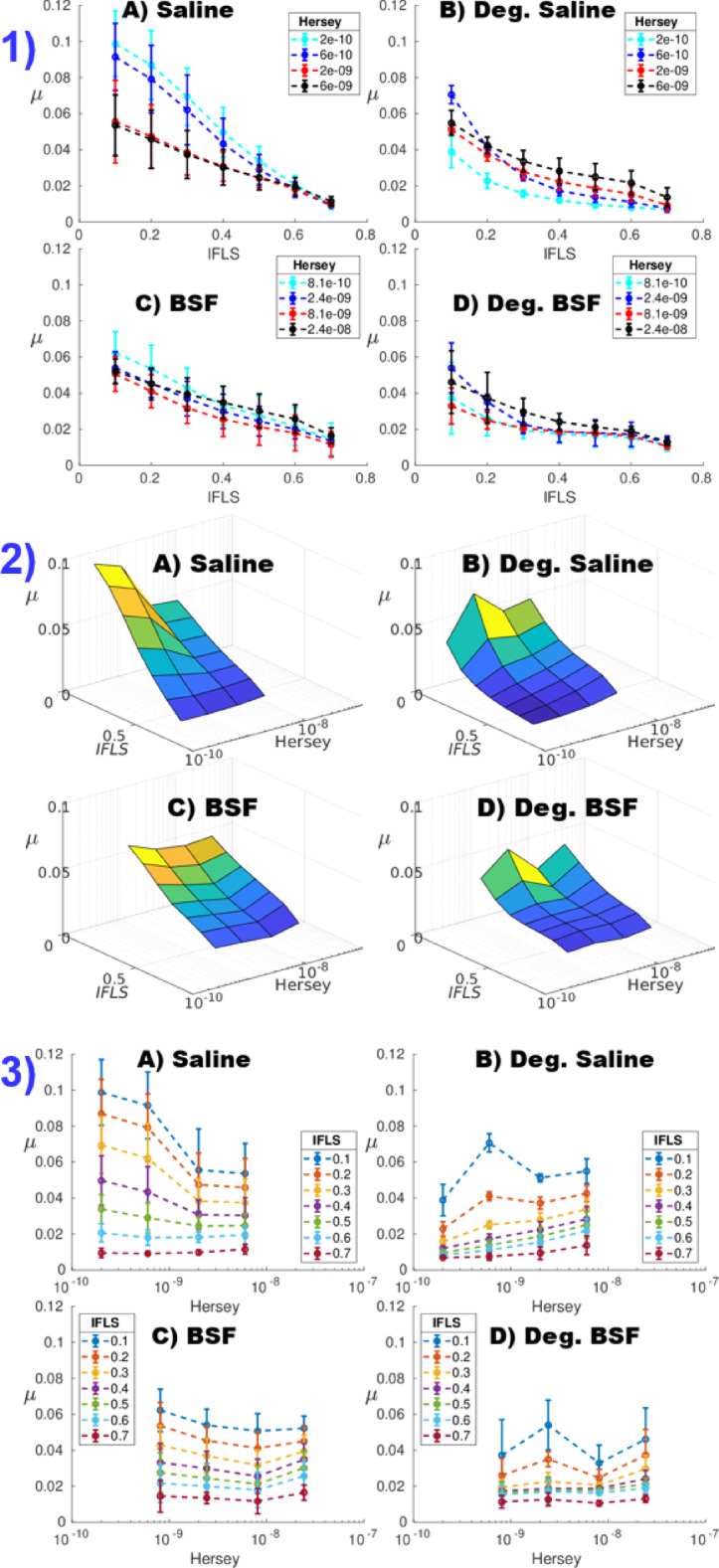
The effect of interstitial fluid load support (IFLS) on coefficient of friction (*µ*). (1) *µ* as a function of IFLS at different Hersey numbers. (2) 3D Stribeck surfaces - *µ* as a function of IFLS and Hersey number. (3)* µ* as a function of Hersey number at different IFLS. Error bars are standard deviation. (**A**) Healthy plugs in saline, (**B**) degraded plugs in saline, (**C**) healthy plugs in bovine synovial fluid, and (**D**) degraded plugs in bovine synovial fluid.

The experimentally measured COF varied non-linearly as a function of the measured axial creep strain for the degraded samples (Fig. 4). Friction regression parameters *µ*_IFLS_ (IFLS-dependent COF), and *µ*_0_ (equilibrium COF) (Table 1) were influenced by the type of lubricant (saline and BSF groups significantly different, *p* = 0.0003 and *p* = 0.002, respectively) and the articulation velocity (for *µ*_IFLS_ *p* = 0.002; for *µ*_0_, *p* = 0.007). Linear regression better predicted (K-W, *p* < 0.0001) the *µ*-IFLS relationship for the healthy samples (*R*^2^ = 0.9990 ± 0.0004) than the degraded samples (*R*^2^ = 0.9956 ± 0.0015), which had a greater degree of non-linearity (Fig. 5 − 1).

**Table 1:** Friction as a function of IFLS. Linear regression model parameters (±standard deviation) for the *μ*-IFLS relationships (Fig. 5) for all the articulation velocities. BSF = bovine synovial fluid, Deg.=degraded, i.e., chondroitinase ABC treated group.

$$\mu = \mu _{{{\mathrm{IFLS}}}} \cdot{\mathrm{IFLS}} + \mu _{0}$$				
Effective velocity	1.5 mm/s	4.4 mm/s	15 mm/s	44 mm/s
**Saline**				
*μ*_*IFLS*_	−0.15 ± 0.03	-0.14 ± 0.03	-0.08 ± 0 04	-0.07 ± 0.02
*μ*_0_	0.11 ± 0.02	0.1 ± 0.02	0.06 ± 0.03	0.06 ± 0.02
*R* ^2^	0.9987 ± 0.0002	0.9989 ± 0.0004	0.9993 ± 0.0002	0.9993 ± 0.0001
**BSF**				
*μ*_*IFLS*_	-0.08 ± 0.03	-0.07 ± 0.01	-0.07 ± 0.01	-0.06 ± 0.01
*μ*_0_	0.07 ± 0.02	0.06 ± 0.01	0.06 ± 0.01	0.06 ± 0.01
*R* ^2^	0.9984 ± 0.0006	0.9989 ± 0.0004	0.9992 ± 0.0004	0.9991 ± 0.0003
**Deg. Saline**				
*μ*_*IFLS*_	-0.08 ± 0.02	-0.13 ± 0.02	-0.08 ± 0.01	-0.07 ± 0.01
*μ*_0_	0.05 ± 0.01	0.09 ± 0 01	0.06 ± 0	0.06 ± 0.01
*R* ^2^	0.9936 ± 0.0006	0.9953 ± 0.0013	0.9963 ± 0.001	0.9966 ± 0.0014
**Deg. BSF**				
*μ*_*IFLS*_	-0.04 ± 0.04	-0.08 ± 0.03	-0.03 ± 0.02	-0.06 ± 0.02
*μ*_0_	0.04 ± 0.02	0.06 ± 0.02	0.03 ± 0.01	0.05 ± 0.02
*R*^2^	0.9945 ± 0.0017	0.9956 ± 0.0012	0.9965 ± 0.0012	0.9964 ± 0.0019

**Fig. 6:**
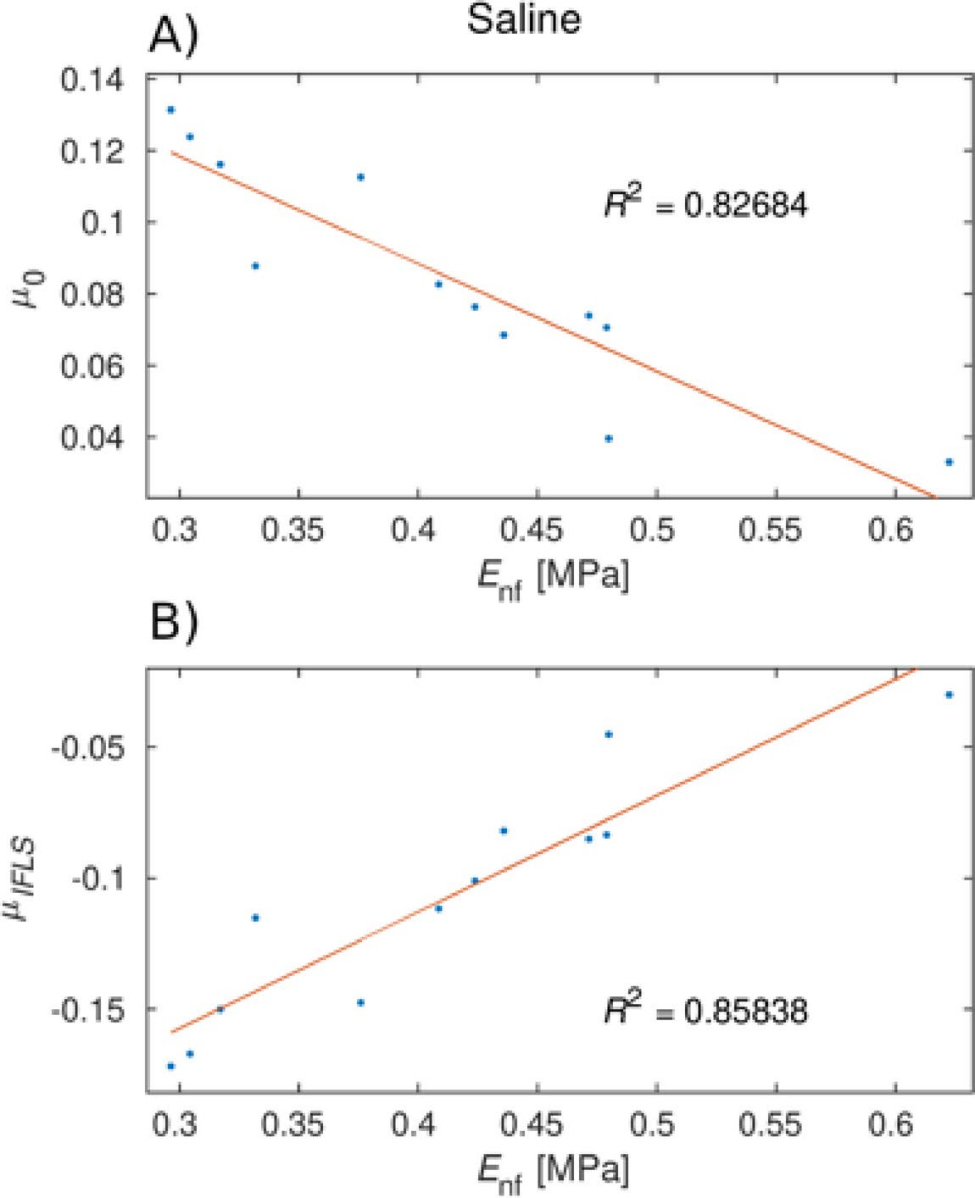
Pearson correlation between friction and nonfibrillar modulus *E*_nf_ for healthy plugs in saline, and the coefficients of determination (*R*²): (**A**) COF at creep equilibrium (*µ*_0_), and (**B**) IFLS dependent COF (*µ*_IFLS_).

According to the regression models, *µ*_IFLS_ was significantly lower for degraded samples compared to healthy samples, for IFLS between 0.25 and 0.7 (Table 1; Fig. 5− 1, 3). Further, *µ*_IFLS_ was significantly lower for BSF relative to saline, for IFLS < 0.35, but significantly higher when IFLS > 0.7. For each articulation velocity (Hersey number), the COF increased with decreasing interstitial fluid load support (Fig. 5 − 1, 3). The only FE parameter to correlate with friction properties was nonfibrillar matrix modulus *E*_nf_ which correlated negatively and positively with *µ*_0_ and *µ*_IFLS_, respectively, for healthy cartilage in saline (Fig. 6).

## Discussion

The study presents a framework for evaluating the coefficient of friction for deformable, poroelastic, biphasic materials such as articular cartilage. The findings consider joint loading characteristics (articulation velocity, applied load, contact area), lubricant (synovial fluid) viscosity, and tissue-specific interstitial fluid load support (IFLS, derived empirically by FE analyses). The highly correlated linear relationship between creep strain and FE derived IFLS (Fig. 3A) concurs with direct experimental measurements of IFLS^21^, validating our approach of calculating the IFLS. The slight difference in the least-mean-square fit linear coefficients reported by Krishnan (*ε* =-0.38·IFLS + 0.34) compared to the present work (*ε* =-0.29·IFLS + 0.34) likely reflects important experimental differences in our study: (a) higher applied compressive traction (780 kPa vs. 89.5 kPa); and, (b) intact osteochondral samples excised from bovine femoral condyle vs. bovine humerus chondral specimens with 2 mm of tissue removed from the deep zone, thereby altering the zonal collagen architecture and influencing the tissue deformation^22^. While linear regression was used for consistency across groups, the samples displayed a degree of nonlinearity (Fig. 3A), suggesting that their behavior is not fully captured by a linear model.

In addition to the magnitude of the applied load, articulation velocity also contributes to the equilibrium creep strain values (*p* = 0.03, Fig. 3B). Burris et al. have described how sliding enhances fluid transport into the tissue^23,24^, and specifically, the external pressure of the flowing lubricant sustains partial fluid load support inside the tissue. Based on their results, sliding speeds > 10 mm/s start affecting tissue compression^25^. Although the tribo-rheometry configuration does not produce hydrodynamic lubrication in a sense where entrained fluid would pressurize the wedge and promote separation of the surfaces, the nonfibrillar modulus *E*_nf_ (the only FE model parameter) differed across articulation velocities (*p* = 0.03), suggesting articulation velocity can influence the apparent nonfibrillar response via fluid pressurization.

By extrapolating the interdependence of axial strain on IFLS and COF on axial strain, we created 3D surface plots expressing cartilage COF as a bivariate function of Hersey number and IFLS (Fig. 5 − 2). This method accounts for 99% of COF variability and allows for the efficient analysis of the effect of material properties, lubricating medium, and applied load conditions on the COF, which would be impossible using compressive strain alone (which depends on the temporal and spatial magnitude of applied tissue deformation). IFLS was found to contribute prominently to the dependence of COF on articulation conditions, with variation in COF as a function of IFLS being greater than that as a function of articulation velocity. The direct relationships between COF and IFLS (Fig. 5 − 1, Table 1) for healthy cartilage are similar to the findings of Accardi et al.^26^ for normal porcine knee cartilage articulating against steel lubricated in PBS (*µ* = -0.16·IFLS + 0.20), and Krishnan et al.^21^ for normal bovine humeral head cartilage articulating against glass lubricated in PBS (*µ* = -0.265·IFLS + 0.270). These findings confirm the significance of tissue pressurization in lubrication. For IFLS < 0.35, COF was lower in BSF than in saline (Fig. 5–1 A, C, respectively), implying that the role of the lubricant becomes more important as the interstitial tissue fluid pressure dissipates. Gleghorn and Bonassar have also noted that lubrication is strongly influenced by tissue pressurization, and that the dissimilarities in friction between various lubricants, such as phosphate buffered saline, equine synovial fluid, and bovine synovial fluid, amplify when cartilage is not under pressurization^27^.

Interestingly,* E*_nf_ influenced the IFLS-dependent friction parameters, decreasing *µ*_0_ and increasing* µ*_IFLS_ (Fig. 6). This correlation was only present for healthy samples and not observed with degraded samples having lower *E*_nf_. While this finding warrants further investigation, it suggests that tissue integrity, potentially related to fixed charge density, modulates cartilage’s tribological behavior.

Chondroitinase treatment induces depletion of cartilage GAGs, resulting in concomitant changes in cartilage structure and function that mimic the material property changes observed in Outerbridge grade 1 cartilage, such as a softer ECM and higher porosity. Proteoglycan depletion also reduces the swelling-pressure–mediated pretension of the collagen network, which can lower the apparent fibrillar stiffness even without direct collagen degradation^28–30^. The FE model parameters, derived empirically for individual specimens, reflect these changes (Fig. 2): decrease in nonfibrillar matrix modulus (*E*_nf_), initial (*E*_f_^0^) and strain-dependent fibrillar network modulus (*E*_f_^ε^), and an increase in permeability (*k*_0_). The type of lubricant, either saline or BSF, did not affect the FE model parameters. Model parameters for degraded specimens exhibit up to a threefold change in decreased nonfibrillar ECM stiffness and increased interstitial fluid flow, consistent with those measured for osteoarthritic human hip joint cartilage^31^. For degraded specimens, the reduced stiffness led to greater tissue strains, but the linear relationship between IFLS and *ε* remained unchanged (Fig. 3A). However, we observed that the COF varied more non-linearly with strain (Fig. 4) and IFLS (Fig. 5 − 1), in contrast to the linear relationship between COF and IFLS reported previously in healthy cartilage^16,21^. Basalo et al.^32^ also observed that chondroitinase-treated samples exhibited frictional responses similar to healthy controls under stress-relaxation testing. In contrast, our findings demonstrate that cartilage degeneration disrupted the IFLS–COF relationship under OA-like conditions during creep articulation, even though the strain–IFLS link remained intact. This non-linearity suggests that as the GAG depletion increases tissue porosity and permeability, it disproportionately accelerates the efflux of interstitial fluid and the depletion of IFLS. When examining IFLS as a function of time at different articulation velocities, degraded samples showed more rapid depletion than healthy ones at low velocities (Supplementary Fig. 2), consistent with their increased permeability (Fig. 2). Importantly, when IFLS remained high, *µ* values were comparable in healthy and degraded cartilage (Fig. 5), reflecting the ability of fluid pressurization to separate the articulating surfaces, much like the air cushion in an air-hockey table. However, because degraded tissue loses this pressurization more quickly, it transitions sooner to higher friction. Because the FE model assumes a constant nominal contact area, solid contact stress is directly proportional to (1 − IFLS), and thus the observed friction–IFLS relationship reflects macroscale biphasic load sharing rather than microscopic contact evolution. At higher velocities, IFLS behavior converged between groups, suggesting that articulation speed compensates for increased permeability and diminishes differences in depletion dynamics. A recent study by Kupratis et al. similarly reported that enzymatically degraded cartilage can retain near-physiological tribological function under sliding, further underscoring that altered solid matrix properties alone do not necessarily diminish lubrication performance^33^.

There are limitations that should be noted for this study. First, the sample size was modest, which may limit statistical power. Second, we did not incorporate the depth-dependent properties of articular cartilage in the FE model. However, previous study has found that FE models incorporating homogeneous and depth-dependent fibrillar structures have comparable IFLS values^34^, providing confidence in our approach. The FE model was formulated to reproduce the axial creep response used for parameter fitting and IFLS extraction. While torsional motion was included experimentally, shear-related dynamic effects (e.g., transient shear-driven fluid flow) were not explicitly modeled. This simplification is unlikely to affect the derived axial material parameters or IFLS estimates, but is acknowledged as a modeling limitation. We also recognize that joint surfaces are not always flat and can exhibit convex or concave geometries. While increasing the sample size aids in attenuating the impact of minor fluctuations in joint surface curvature, particularly if these variations occur randomly, it’s essential to acknowledge that considering a flat interface and using an averaged cartilage thickness constitute modeling simplifications. Consequently, more pronounced geometrical irregularities that could locally affect load distribution and friction are not captured by the present approach. In addition, representing two articulating cartilage layers as a single effective layer with averaged thickness is a further approximation adopted for modeling the combined creep response of the plug pair. It is also important to note that cartilage tissue reaches equilibrium faster in an unconfined geometry, whereas in physiological sliding against cartilage, a migrating contact area is generated able to maintain elevated interstitial fluid load support. Finally, the FE model did not simulate the brief lift-off periods used in the experiment, during which rapid rehydration and transient pore-pressure recovery occur. While our analyses suggest that incorporating such lift-offs does not alter the fitted material parameters obtained from the optimization procedure, they do influence the temporal IFLS profile in a manner that depends on the degree of tissue degeneration. Thus, both the modeled pressurization and the exact IFLS values derived from it should be interpreted with appropriate caution. More generally, all reported material parameters in this study should be understood as model-dependent fitting parameters representing bulk tissue behavior rather than directly measured intrinsic tissue properties.

Looking ahead, more biomimetic models could be developed to better reflect the complexity of in vivo joint mechanics and improve our understanding of the mechanisms governing IFLS. This includes incorporating swelling behavior^35^, mechanobiological effects^36,37^, and depth-dependent variations in the chemical composition and micro-structural anatomy of the tissue^38–40^. Additionally, replicating loading scenarios relevant to the femoral condyles during activities such as walking, running, jumping, or traumatic injury could further elucidate the relationships among tissue permeability, strain, and fluid load support.

Together, these findings highlight interstitial fluid load support as a unifying principle that links cartilage structure, mechanics, and lubrication. Beyond clarifying how degeneration alters this balance, our combined tribological–modeling approach provides a quantitative framework to assess how tissue repair or engineered constructs restore fluid pressurization. The identification of an IFLS threshold further suggests a potential diagnostic marker to evaluate early degeneration or the success of therapeutic interventions. More broadly, the methods and concepts presented here may be extended to other biphasic and viscoelastic systems, including hydrogels and engineered joint replacements, where fluid–solid interactions govern durability and function. By bridging experimental testing with computational modeling, this work opens pathways for translating cartilage tribology into predictive, clinically relevant measures of joint health.

## Methods

### Samples

Osteochondral plug explants obtained from a local slaughterhouse (*N* = 24, 7 mm diameter, > 1 cm of subchondral bone) were extracted from the medial and lateral femoral condyles of mature (estimated 18–24 months; exact age and sex unknown) bovine knees using a diamond tipped cylindrical coring tool (Starlite Industries, Bryn Mawr, PA). Only femoral-condyle plugs were used to reduce geometric and material variability, as both human and animal studies indicate these sites are more uniform and mechanically consistent than the tibial plateau^41,42^. As no animals were sacrificed specifically for this research, ethical approval was not required. Only normal cartilage tissue (Outerbridge grade 0) was harvested. Plugs were stored at -20 °C in 400 mOsm sodium chloride solution containing protease inhibitor benzamidine hydrochloride (5 mM), GIBCO Antibiotic/Antimycotic (Invitrogen, Grand Island, NY), and calcium ion chelating agent ethylenediamine tetraacetic acid (5 mM).

Before experiments, the sample tubes were thawed in a water bath at room temperature (~ 22 °C) for one hour. To allow fluid redistribution within the tissue, each sample pair was preconditioned under a static compressive preload of 0.2 MPa for 2 h. Half of the plugs were enzymatically degraded in 1 mL of 0.5 U/mL Chondroitinase ABC (Sigma Aldrich, St. Louis, MO) in PBS at 37 °C for 24 h, followed by thorough PBS rinsing^32^. All samples were subsequently incubated for 24 h at 4 °C in their assigned lubricants. Throughout degradation and incubation, the plugs were kept under gentle agitation and fully immersed (≥ 1 mL) to ensure uniform exposure. Baseline cartilage thickness was determined using µCT imaging after the incubation period. Tribological testing was then performed on consecutive days at each velocity, and the samples were allowed to reswell overnight in their assigned lubricants while refrigerated at 4 °C. Finally, µCT imaging was repeated 24 h later to confirm the absence of tissue wear.

### Computed tomography

Cartilage geometry and morphology were measured from reconstructed 3D µCT images (Fig. 1C) at isotropic voxel size of 36 µm^3^ (µCT40, Scanco Medical AG, Brüttisellen, Switzerland) using an airtight sample holder to maintain a humid environment to prevent tissue drying. The µCT data were converted to DICOM format and thicknesses were analyzed using a Matlab (R2016b, The MathWorks, Inc., Natick, MA, USA) script calculating average surface-to-bone distance over the entire cartilage area.

### Tribology

Bi-axial torsional mechanical friction tests (BOSE Electroforce 3200) were conducted on randomly paired, coaxially aligned osteochondral plugs with congruent apposing cartilage surfaces submerged in a lubricant bath (Fig. 1). The specimens were subjected to a constant nominal compressive stress of 0.78 MPa^43^ with a displacement rate of 1 mm/s; simultaneous torsional rotation was applied at four increasing angular velocities (36°/s, 108°/s, 360°/s, 1080°/s), with overnight reswelling between each step. All experiments were performed at room temperature using three paired osteochondral plug sets (six plugs per group), resulting in a total of 24 plugs across four experimental groups. The corresponding effective articulation velocities (tangential speed at the effective radius) were 1.5, 4.4, 15, and 44 mm/s for 10,000 s. The effective radius is calculated by integrating the speed distribution over the circular contact area: *r*_eff_ = (2/3)·*r*, where *r* = plug radius. The applied forces and torques were measured with an in-line 150 N axial load-cell (Honeywell, Columbus, OH) and 0.15 Nm torque-cell (Honeywell, Columbus, OH). At each velocity, alternating rotations (+ 6 revs, -6 revs) were applied for 18, 124, 180, 516 rotations, respectively. This was followed by a lift-off for 10 s (Fig. 1B), to replenish the lubricant between the surfaces, after which testing was resumed and repeated until the end of the last full cycle (174–184 min). Ten-second lift-off events were identified from the force signal and excluded from analysis. The torque data were subsequently filtered using a 10 s moving average window (Fig. 1B).

Lubricants included healthy bovine synovial fluid (BSF, viscosity = 3.5 mPa·s) and 0.9% normal saline (NS, viscosity = 0.9 mPa·s). Osmotic environment is known to alter cartilage biomechanics^44^. Therefore, the osmolality of the saline was adjusted to that of healthy SF: 400 mOsm^45,46^. Four groups of osteochondral plugs were tested: (1) Outerbridge 0 lubricated with BSF, (2) Outerbridge 0 lubricated with saline, (3) Outerbridge 1 lubricated with BSF, (4) Outerbridge 1 lubricated with saline. Engineering strain and creep were analyzed from the axial deformation data. Coefficient of friction was calculated from *µ* = (3/2) · (*τ* / *Nr*), where: *τ* = torque (measured @ 10 Hz), *N* = load^47^. Measured torque amplitudes were well above baseline instrument noise. Although short-term torque fluctuations were observed during testing, friction parameters were derived from regression over long-duration responses and were not affected by such transient variability.

### FE modeling, IFLS extraction, and statistics

**Fig. 7:**
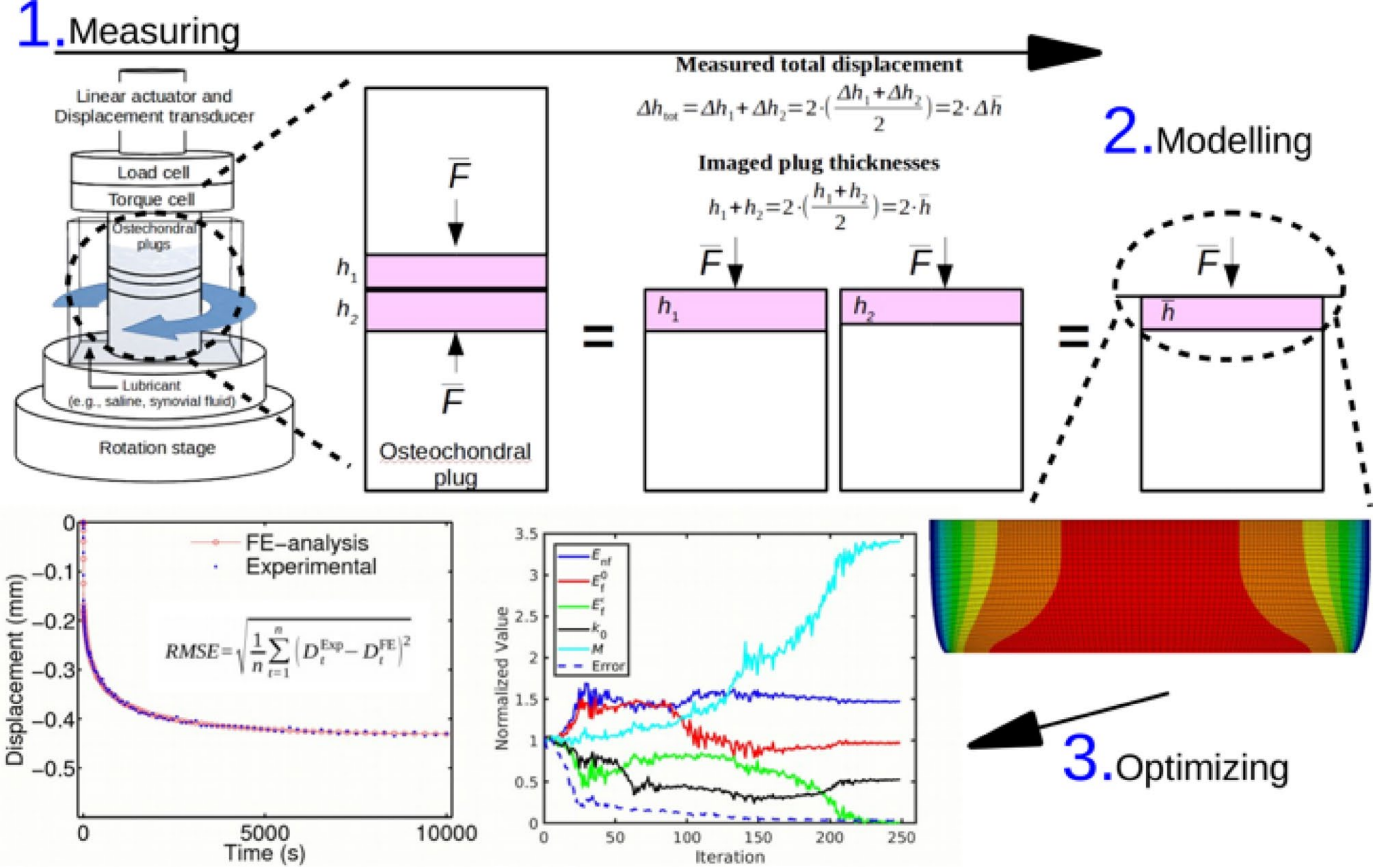
Representative illustration of the optimization process. Fibril-reinforced poroviscoelastic model (shown mirrored for visual clarity) is employed to replicate average experimental strain of two plugs from biomechanical testing. Model concordance to the experimental strain is achieved with minimizing cost function (RMSE, Error) by optimizing model parameters: initial and strain-dependent fibril network modulus (*E*_f_^0^, *E*_f_^*ε*^, respectively), nonfibrillar matrix modulus (*E*_nf_), initial permeability (*k*_0_) and strain-dependency of permeability (*M*).

To extract the behavior of the IFLS during experimentation, an axisymmetric fibril-reinforced poroviscoelastic FE creep model was created (Abaqus 6.13; Dassault Systèmes Simulia Corp). The design is able to accurately replicate articular cartilage inhomogeneity and anisotropy in biomechanics and it has been well-validated in the past^19,48^. While absolute material values may depend on loading configuration, they remain consistent within the unconfined creep protocol employed in this study^49^. The total stress tensor represents the combined contributions from the fibrillar and nonfibrillar matrices and the pore fluid pressure^29,50^. Consistent with our previous work, the fibrillar phase was modeled as a tension-only viscoelastic collagen network with linearly strain-dependent stiffening, the nonfibrillar solid matrix as a Neo-Hookean hyperelastic material carrying all compressive stresses, and the fluid phase as interstitial flow governed by Darcy’s law with strain-dependent permeability^13^. The model (Fig. 7) assumed: (a) no torsional shear; (b) a constant axial load was applied via an impermeable rigid half-plane; (c) model thickness was the average thickness of the two plugs; (d) representative creep for the model was 50% of the total axial displacement.

The FE mesh consisted of 2,400 linear axisymmetric pore pressure continuum elements (CAX4P) and mesh convergence was verified by confirming that further mesh refinement did not significantly alter the primary outcome variables (pore pressure and derived IFLS). The applied torsional shear was estimated to be negligible compared to applied axial compression. Incongruence between articulating surfaces was observed to be minimal and full contact was estimated at the beginning of the creep. Applied boundary conditions were: (a) the cartilage bone interface was fixed in all directions; (b) the cartilage edges were assumed to be fully permeable; (c) the platen – cartilage, and cartilage – bone interfaces were impermeable; (d) along the axis of symmetry, lateral displacements were prevented, and fluid could not penetrate this boundary. The viscoelastic fibril reinforced network comprised of primary fibrils aligned parallel to the cartilage surface, and secondary fibrils oriented randomly throughout the model^3,20,40^. The only structural property that was sample specific was cartilage thickness, which was measured for each plug and implemented as the average of the two articulating plugs in the model (Fig. 7). Except for the optimized model parameters, the model structure and fixed model constants were homogeneous and equivalent for both healthy and degraded samples. Structural properties were not measured for the samples, so estimating and implementing sample specific or depth-dependent collagen orientation, collagen content and/or GAG content in the models would have led to deceptive estimations in turn making the modeling analysis more dependent on the structure.

Model concordance to sample specific experimental measurements was achieved by minimizing the root-mean-square error between the measured axial deformation and the calculated model displacement^51^(Fig. 7) by adjusting the following parameters: (a) initial and strain-dependent fibril network modulus, *E*_f_^0^, *E*_f_^ε^, respectively, (b) nonfibrillar matrix modulus, *E*_nf_, (c) initial permeability, *k*_0_, and strain-dependent permeability factor, *M*. *E*_f_^0^describes the stiffness of the fibrillar network when there is no deformation, while *E*_f_^ε^ describes how the stiffness changes as the tissue is deformed. *E*_nf_ represents the stiffness of the part of the tissue that is not reinforced by collagen fibrils. *k*_0_represents the permeability of the tissue at zero strain or stress. It is a measure of how easily fluid can flow through the tissue under initial loading conditions. On the other hand, *M* is a measure of how much the tissue’s permeability changes in response to deformation or compression.

**Fig. 8:**
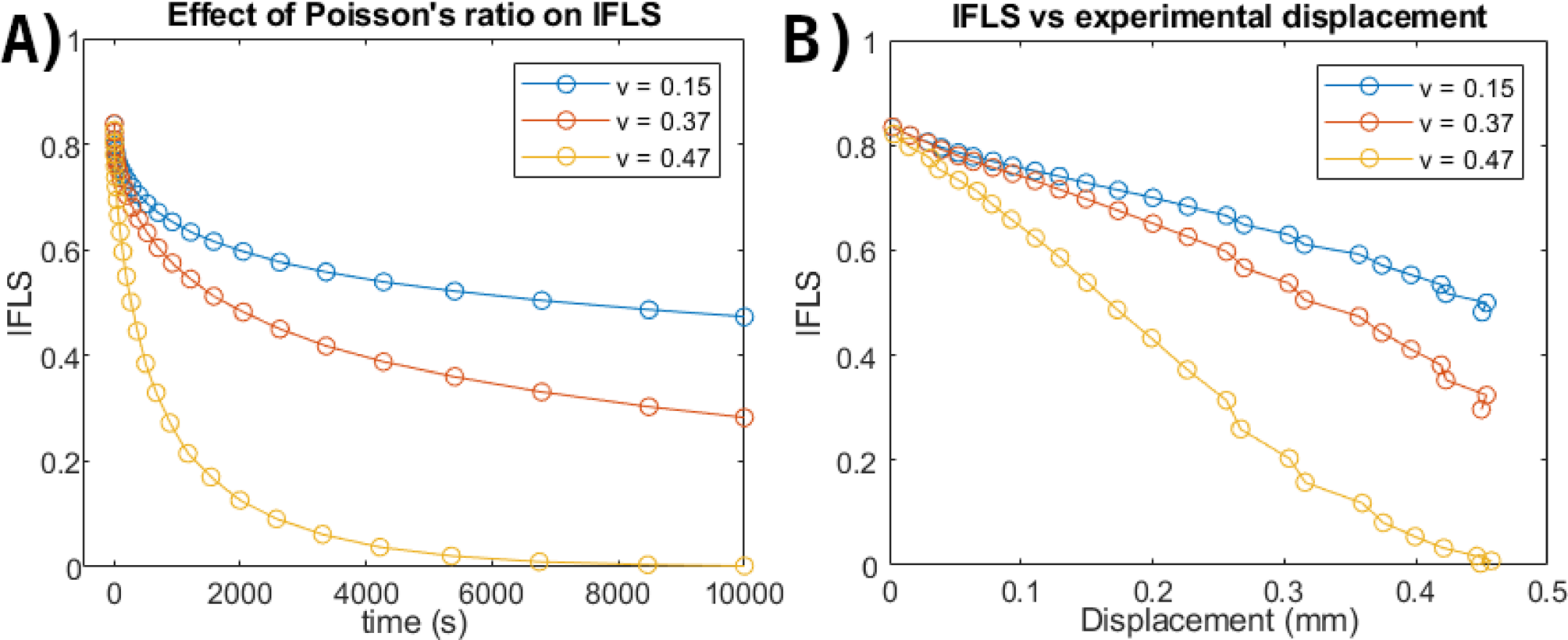
Sensitivity of the model to the nonfibrillar matrix Poisson’s ratio (*ν*). (**A**) IFLS as a function of time for *ν* = 0.15, 0.37, and 0.47, with all other optimized parameters held constant. (**B**) IFLS as a function of experimental displacement for the same parameter sets.

The following parameters were set as constants to consolidate uniqueness of the optimization results and were based on experimental works where they have been successfully used in representing the behavior of human and bovine articular cartilage: damping coefficient of the viscoelastic fibrils (*η*) was set to constant 947 MPa·s^3,20,52^, Poisson’s ratio of the nonfibrillar matrix was fixed to 0.47^53,54^, and initial void ratio was set to 3.5 (equaling to a water fraction of 78%)^55,56^. The Poisson’s ratio specified in the model applies only to the nonfibrillar matrix. Due to the coupled fibrillar–poroviscoelastic formulation, the apparent tissue-level Poisson-type response is governed primarily by the fibrillar network rather than by the nonfibrillar matrix alone, as demonstrated previously^57^. Therefore, the assigned nonfibrillar matrix Poisson’s ratio should not be interpreted as a direct measure of experimentally reported tissue-level Poisson’s ratios. To examine the sensitivity of the modeled response to the fixed Poisson’s ratio parameter, additional simulations were performed with *ν* = 0.15, 0.37, and 0.47 while keeping all other optimized parameters fixed (Fig. 8). Across the tested range, *ν* influenced the absolute magnitude and dissipation rate of IFLS, whereas the overall relaxation behavior and displacement dependence were preserved. The optimization results were verified by altering the initial values and constantly obtaining the same results. The same parameters as optimized here have also been successfully optimized in earlier studies^8,29,53^. Thus, unique material parameters should have been obtained from each optimization. The average pore pressure (POR) was extracted from the model and IFLS = POR/contact pressure was calculated. Contact pressure was defined as load/area and not computed through the contact algorithm.

### Statistics

Linear regression was used to study *µ*-IFLS relationship:

1 $$\mu = \mu _{{{\mathrm{IFLS}}}} \cdot{\mathrm{IFLS}} + \mu _{0} ,$$

where *µ*_IFLS_ is the IFLS dependent COF; *µ*_0_ is the COF at creep equilibrium, when IFLS = 0, i.e., *µ*(0).

Results are presented as means with standard deviation. Coefficient of friction (*µ*) results are presented as a function of the effective articulation velocity, and finally combined using a Hersey number^58^. Hersey number allows to tie the friction to a dimensionless lubrication parameter (i.e., articulation velocity · lubricating fluid viscosity / load per unit length). The Hersey numbers, corresponding to the lubricants and test conditions, derived by multiplying velocity [m/s] with viscosity [Pa·s], and dividing by load per unit length [N/m], were 8.1·10^–10^, 2.4·10^− 9^, 8.1·10^− 9^, 2.4·10^− 8^, respectively. Owing to viscosity differences, the range of Hersey numbers between saline and BSF groups were different. Therefore, to allow statistical comparisons between the groups, the effective velocity is used as the common denominator. Two-sided statistical tests were performed using Matlab, with *p* < 0.05 significance threshold. Post-hoc Kruskal-Wallis (K-W) test was used to study intergroup differences, based on non-parametric data distribution (Kolmogorov-Smirnov test). Friedman test was used to study the effect of velocity on the results. Pearson correlation was used to study the effect of FE model parameters on *µ*_0_ and *µ*_IFLS_.

Rather than varying the articulation velocity randomly, measurements were initially conducted at the slowest velocities and increased sequentially, progressing from low velocity to higher velocities, which could have introduced systematic error and bias as to the independent effects of articulation velocity on COF. In contradistinction to the healthy samples, the *µ*_IFLS_ values for the degraded specimens reduced at the lowest articulation speeds. There exists the possibility that chondroitinase released surface-active proteins from inside the tissue (e.g., phospholipids, lubricin, or hyaluronic acid) during the first, i.e. the lowest velocity, measurements. Because no thorough washout was performed before immersing the samples in lubricant, these molecules may have temporarily altered the lubrication environment and measurement conditions^59,60^. To avoid confounding effects, results from the slowest velocity experiments were excluded from statistical group comparisons.

## Supplementary Information

Below is the link to the electronic supplementary material.

Supplementary Material 1

## Data Availability

The datasets used and/or analyzed during the current study available from the corresponding author on reasonable request.
