# Supplementary material for: Exploring mechanisms governing cartilage interstitial fluid load support in lubrication through experimental and computational analysis
Source: Sci Rep. 2026 Mar 10;16:12902. doi: 10.1038/s41598-026-41939-9 (PMC13096607; doi:10.1038/s41598-026-41939-9)
Supplement: Supplementary file 1 — Supplementary Material 1 [file 41598_2026_41939_MOESM1_ESM.docx]

SUPPLEMENTARY INFORMATION

**Exploring Mechanisms Governing Cartilage Interstitial Fluid Load Support in Lubrication through Experimental and Computational Analysis**

**Janne T.A. Mäkelä^1,2,3*^, Taylor Lawson^1,4^, Rami K. Korhonen^3^, Mark W. Grinstaff^2,5,6*^, Brian D. Snyder^1,7*^**

^1^Center for Advanced Orthopaedic Studies, Beth Israel Deaconess Medical Center, Harvard Medical School, Boston, MA, USA

^2^Department of Chemistry, Boston University, Boston, MA, USA

^3^Department of Technical Physics, University of Eastern Finland, Kuopio, Finland.

^4^Department of Mechanical Engineering, Boston University, Boston, MA, USA

^5^Department of Biomedical Engineering, Boston University, Boston, MA, USA

^6^Department of Medicine, Boston University, Boston, MA, USA

^7^Department of Orthopaedic Surgery, Boston Children’s Hospital, Harvard Medical School, Boston, MA, USA

*****Co-correspondence

**Janne T.A. Mäkelä**

University of Eastern Finland

*Janne.Makela@uef.fi*

**Mark W. Grinstaff**

Boston University

*mgrin@bu.edu*

**Brian D. Snyder**

Boston Children’s Hospital

*Brian.Snyder@childrens.harvard.edu*

**Author Contributions**

All authors contributed to the conception and design of the study, and interpretation of the results. Data was generated by JTAM and TBL, which was analyzed and interpreted by JTAM. Manuscript was prepared by JTAM with the help of other authors. RKK, MWG and BDS provided supervision of the project, and editing. JTAM takes responsibility for the integrity of the work.


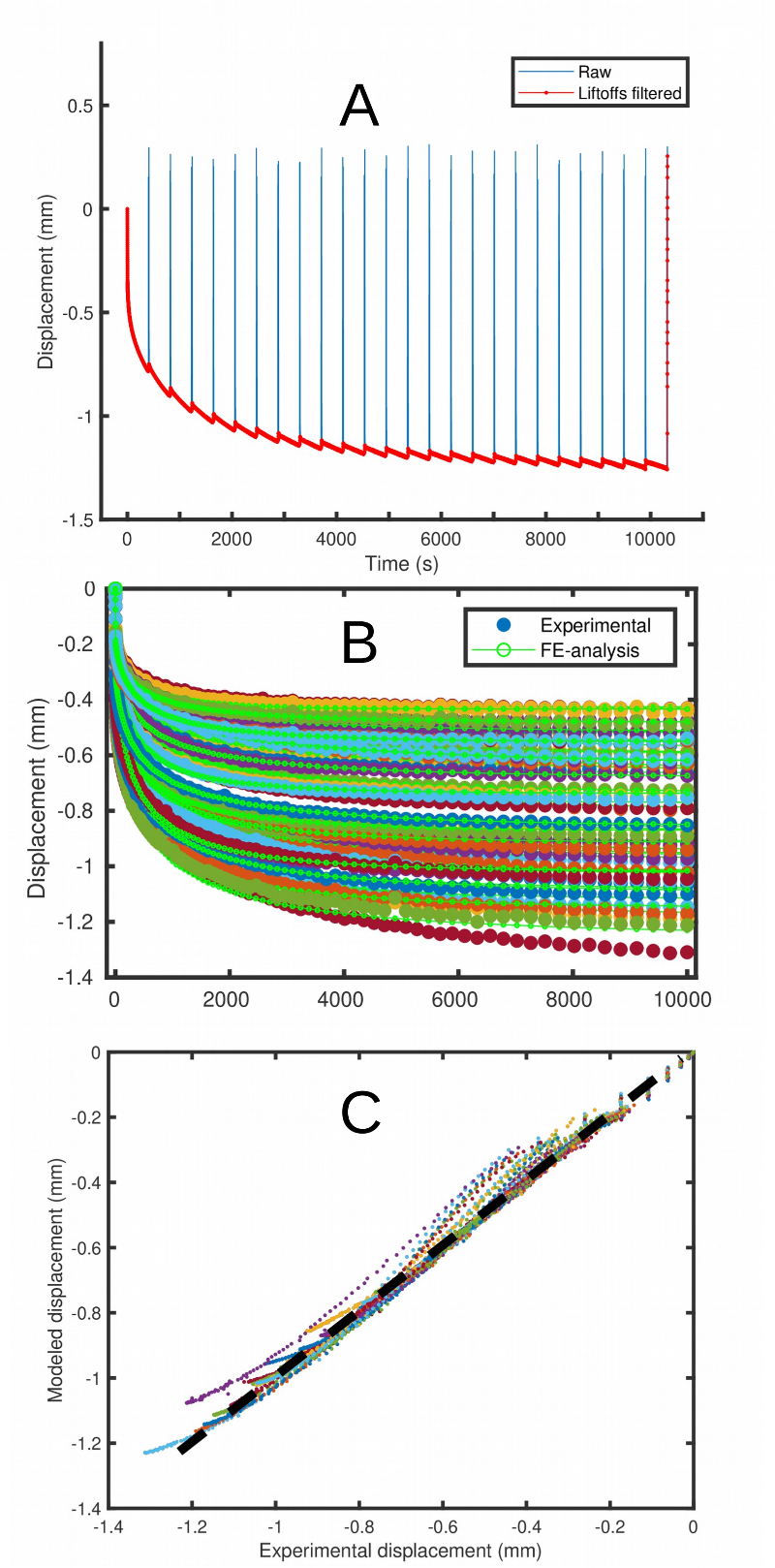


**Supplementary Fig. 1:** Experimental and modeled displacement results. A) Raw and filtered (lift offs removed) data measured experimental displacement. B) Experimental vs Finite element (FE) model displacement creep curves as a function of time. C) The experimental vs FE model displacement. Dashed line represents the 1:1 curve.


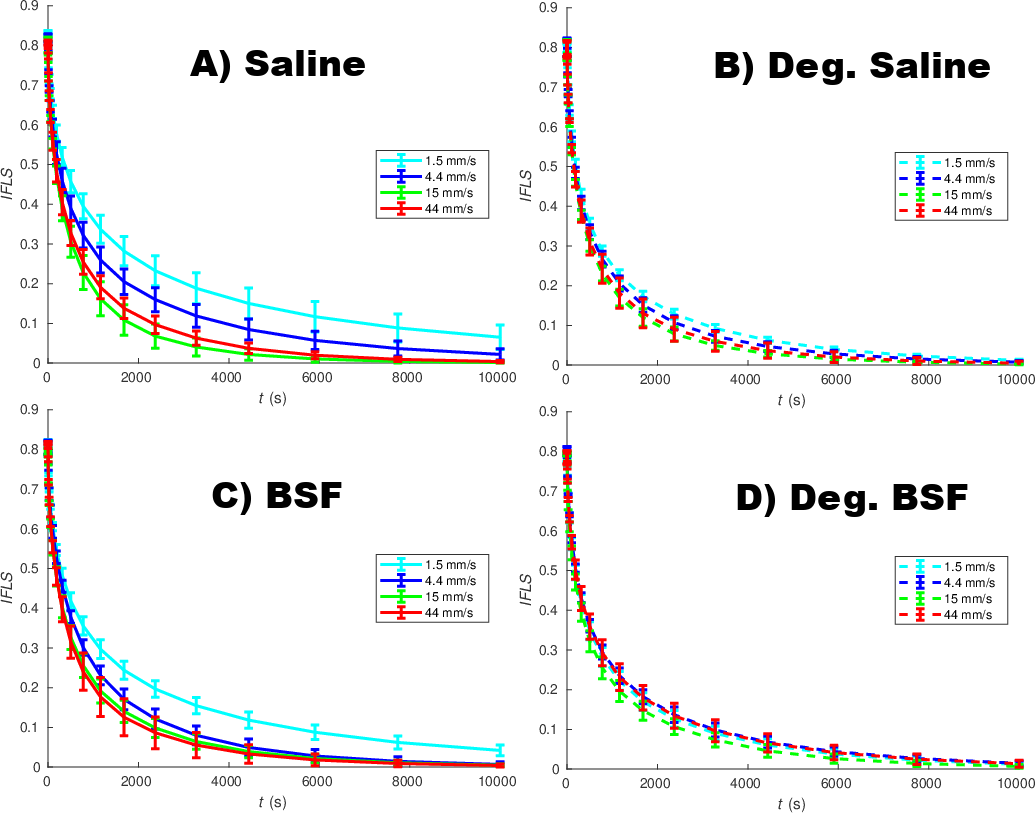


**Supplementary Fig. 2:** Average interstitial fluid load support (IFLS) as a function of time at different effective velocities (*v*_eff_=1.5, 4.4, 15, 44 m/s). A) Healthy plugs in saline, B) degraded plugs in saline, C) healthy plugs in bovine synovial fluid, and D) degraded plugs in bovine synovial fluid. Extracted from the modeling results. Error bars mark standard deviation.
